# Atomistic Study of the Effect of Hydrogen on the Tendency toward Slip Planarity in Bcc Iron

**DOI:** 10.3390/ma16144991

**Published:** 2023-07-14

**Authors:** Yuanyuan Zheng, Gang Zheng, Kaiyu Zhang, Lili Cao, Ping Yu, Lin Zhang

**Affiliations:** 1 School of Mechanical and Energy Engineering, Zhejiang University of Science and Technology, Hangzhou 310023, China; 222201855029@zust.edu.cn (G.Z.); lilytaurus@zust.edu.cn (L.C.); 2 Institute of Material Forming and Control Engineering, Zhejiang University of Technology, Hangzhou 310014, China; 2111825057@zjut.edu.cn (K.Z.); zhlin@zjut.edu.cn (L.Z.); 3 School of Materials Science and Engineering, Shanghai Jiao Tong University, Shanghai 200240, China

**Keywords:** dislocation, binding energy, hydrogen, cross-slip, slip planarity

## Abstract

H-enhanced slip planarity is generally explained in terms of H-reducing stacking fault energy in fcc systems. Here, we showed that H-decreasing dislocation line energies can enhance the tendency toward slip planarity in bcc Fe through systematically studying the interaction between H and 1/2 <111> {110} dislocations using the EAM potential for Fe-H systems. It was found that the binding energy of H, the excess H in the atmosphere, and the interaction energy of H increased with edge components, leading to larger decrements in the line energies of the edge and increased mixed dislocations than those of a screw dislocation. The consequence of such interaction patterns is an increment in the energy change in the system when the edge and mixed dislocations are converted to screw dislocations as compared to the H-free cases. The cross-slip in bcc Fe is thus suppressed by H, increasing the tendency toward slip planarity.

## 1. Introduction

Hydrogen embrittlement (HE) has been a long-standing, unresolved problem for the safe use of almost all hydrogen-based materials [1,2,3]. Despite extensive studies over the past century, a complete understanding of HE is still lacking. It is now generally accepted that the activity of hydrogen with dislocations is crucial to the embrittlement process [4,5]. Metallographic and fractographic observations have provided evidence that the nucleation and emission of a dislocation from a crack tip is enhanced by hydrogen. In situ deformation experiments using environmental cell transmission electron microscopy (TEM) have revealed that H enhances dislocation motion, decreases stacking fault energy, and stabilizes edge dislocations [6]. Stress relaxation tests have demonstrated that H decreases the activation volume of mobile dislocations and increases their dislocation velocity and density. TEM observations using samples extracted from fracture surfaces by focused ion beam (FIB) machining have suggested that H-induced flat fracture surfaces are closely related to dense local dislocation activities [7,8,9].

There have been extensive studies on H-dislocation interactions to develop an understanding of how H affects dislocation behaviors. For example, based on experimental measurements of diffusivity, internal friction, and enhanced solubility or permeability, H binding energies with dislocations have been estimated for various materials [10,11]. Atomistic simulations have provided details about single-H binding at dislocation cores [12,13] and the distribution of H around dislocations [14]. Moreover, elastic theory has also been used to estimate the interaction between H and dislocations in a region far away from the cores [15]. However, most of the previous research has focused only on pure edge or screw dislocations, and there is little research on the mixed dislocations that are also typical defects formed during the deformation process. To the best of our knowledge, only Gesari et al. [16] calculated the H binding energy of 0.22 eV with 72.52° dislocation in bcc Fe, and Wen et al. [17] obtained the H binding energy of 0.10 eV with 30° and that of 0.13 eV with 60° partial cores in Ni. Therefore, there are no complete studies on the interaction between H and dislocations.

One important result of the interaction between H and dislocations revealed by microstructural observations is that H can increase the propensity for slip planarity in 310s austenitic steels, Al, Ni-Co, Ni, and Ni-based superalloys [18,19]. The slip planarity would inhibit the relaxation of dislocation pileups by cross-slip and restrict dislocations from a given source to a narrow slip band. Consequently, a pileup with a larger population of and more closely spaced dislocations can be formed, which could initiate microcracks at the head of the pileup. On the other hand, slip planarity could induce local high H concentrations near the grain boundaries, precipitates, and other obstacles by dislocating the transport of H, facilitating fractures through slip localization, reducing cohesive strength, and voiding growth or the formation of local hydride [1,20]. Thus, slip planarity usually increases the susceptibility of a material to HE. There are two mechanisms that cause H-enhanced slip planarity. The first mechanism applies more often to fcc systems and considers that H can reduce stacking-fault energy (SFE), which increases the equilibrium separation distance between two partial dislocations, and thus, it causes constriction, a critical step that makes cross-slip difficult to occur and, consequently, leads to planar slip [21]. The reduction in the SFE in 310s austenitic steels, AISI 304 steel, and Ni has been measured by experimental methods [22]. The second mechanism can apply to all systems and proposes that H forms an atmosphere primarily around edge dislocations and decreases the energy of a system; however, it does not occur around screw dislocations. This makes edge dislocations more favorable as compared to the H-free case, and therefore, the proportion of screw dislocations is decreased and the tendency to cross-slip is inhibited by H. This mechanism has been supported by in situ TEM observations of the character of dislocations in high-purity Al, and the H-induced reduction in edge dislocation energy in the Al was then calculated based on the elastic theory [23]. It should be noted that from this calculation, the mechanism can only occur at extremely high bulk H concentrations, where *c_0_* ≥ 10,000 appm, which is several orders of the bulk H concentration imposed in experiments. The mechanism of the edge dislocation energy reduction in slip planarity thus needed to be further clarified.

There is, in fact, a lack of studies on the effect of H on slip planarity where dislocations have compact core structures in bcc Fe. In the present study, we studied the effect of H on slip planarity in bcc Fe using the embedded-atom-method (EAM) potential for an Fe-H system. We systematically studied the interaction between H and 1/2 <111> {110} dislocations. The interaction energy of the hydrogen with the dislocations, the trapped H around dislocations, and the effect of the H on the dislocation energies were evaluated and analyzed. The influences of H on the slip planarity in bcc Fe are then discussed.

## 2. Simulation Method

**Figure 1:**
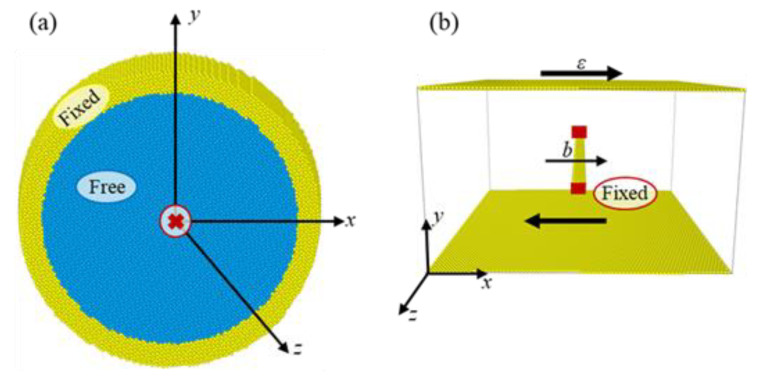
The simulation block showing the dislocation model in this work, where the regions of the fixed atoms and free atoms are marked. (**a**) The simulation model box, (**b**) Configuration of an edge dislocation pinned at the obstacles on either end, acting as a Frank–Read source. Only the non-bcc-coordinated atoms are shown, corresponding to the dislocation core region.

The dislocations were labeled by the character angle *θ* (0° ≤ *θ* ≤ 90°), which we defined as the angle between the dislocation line direction and its Burgers vector. All dislocations were arranged with the same Burgers vector (b=[111]) and slip plane ($n=[\bar{1}10]$). Therefore, the line directions of the dislocations *l* could be calculated by l·n=0 and $sin\theta =\frac{l\;\times \;b}{\left|l|\cdot |b\right|}$, and the obtained results at different *θ* values are listed in Table 1. A cylindrical box with a radius of 10 nm was designed for the atomistic simulations, as shown in Figure 1. The *y*-axis of the box was oriented along $\left[\bar{1}10\right]$, the *x*-axis of the box was parallel to [111], the *z*-axis was parallel to $\left[\bar{1}\bar{1}2\right]$, and the line directions had lengths of three repeated unit cells. The simulation system contained a total of 447,480 Fe atoms. By using this arrangement, the *x*-axis could be determined by x=y×z, and the results are also listed in Table 1. The dislocations were placed at the center of the box by displacing all atoms according to the anisotropic elastic displacement fields. After introducing the dislocations, the atoms inside the outer shell (which had a thickness of 10.6 Å, i.e., two times the cut-off distance of the Fe–Fe interatomic potential), were fixed, and the rest of the atoms were allowed to move freely. The conjugate gradient method was then employed to relax the simulation box to obtain the equilibrium dislocation structures.

The atomic interaction was described by the embedded-atom-method (EAM) potential for the Fe-H system developed by Song et al. [24]. The EAM potential for an Fe-H system was developed based on the first principles (DFT) calculations of a series of single and multiple H properties in Fe. The EAM potential takes into account the interaction energy of a single hydrogen atom in the Fe matrix dissolution, vacancy, and free surface, and the interaction between multiple hydrogen atoms and the vacancy and the activity of the H-H in the Fe matrix are also considered. It can accurately describe the behavior of individual hydrogen atoms in the Fe matrix, and it also reliably describes the behavior of hydrogen atoms in the vicinity of defects (vacancy, dislocation, crack tip, etc.). The EAM potential not only provides accurate H solution energy in Fe and binding energies to edge/screw dislocations but also avoids the unreal clustering of H in bulk Fe, in accordance with the phase diagram for an Fe-H system; therefore, the potential is reliable for describing H properties in Fe.

The Monte Carlo method is suitable for a system with a constant number of atoms, but when the total number of atoms in the system changes, the above method is not applicable. In this case, the Monte Carlo method under the great canonical ensemble, that is, the grand-canonical ensemble Monte Carlo (GCMC), is needed. In a GCMC simulation, the volume *V*, temperature *T,* and chemical potential *μ* of a system remain unchanged, and the total number of atoms of a system can be achieved by inserting/deleting target atoms into a simulation interval. We charged H into the whole simulation box using the GCMC method at 300 K. The bulk H concentration *c_0_* was related to the *μ* of the H as follows: $lnc_{0}=\mu /kT+E^{f}/kT$. Here, *c_0_* = *n_H_*/*n_Fe_*, where *n_H_* is the number of H atoms, *n_Fe_* is the number of Fe atoms, *k* is the Boltzmann constant, and *E^f^* = 2.003 eV/H is the formation energy of H in the dilute limit at 300 K obtained in our previous study [25]. Thus, the equilibrium H distribution around the dislocations could be obtained by specifying the chemical potential *μ*. Each GCMC step consisted of three motion attempts: the insertion of a hydrogen atom at any location in the free region, the deletion of arbitrary hydrogen atoms, and the movement of any atom (hydrogen or Fe) in any direction. The success of atomic movement was determined according to Markov criterion, and the insertion and deletion of hydrogen atoms were determined according to the Adams algorithm. Each GCMC step detected 50 arbitrary locations to determine the location within the pore. The attempted ratios for the displacement, insertion, and deletion moves in our simulations were 50%, 25%, and 25%, respectively.

## 3. Results

### 3.1. H Binding Energy

The binding energy of H with the dislocation *E^bind^* was calculated using molecular statics (MS), and it was defined as:

(1) $$E^{bind}=\left(E^{dis}+E^{H}\right)-(E^{dis+H}+E^{0})$$

where *E^dis^* is total energy of the system containing a dislocation, *E^H^* is the total energy of the system containing one H-atom at a tetrahedral site in an Fe lattice, *E^dis+H^* is the total energy of a system containing a dislocation and one H-atom, and *E^0^* is the total energy of the perfect Fe bulk. With such a definition, a positive binding energy would indicate attraction between an H-atom and the dislocation.

**Figure 2:**
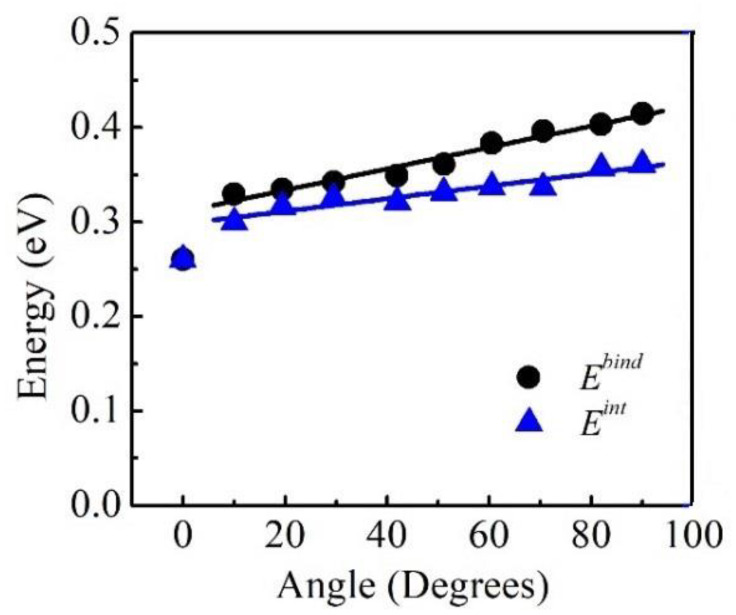
H Binding energy *E^bind^* and the interaction energy *E^int^* as a function of *θ*.

The obtained binding energies of the H-atoms are shown in Figure 2, where it is clearly seen that the *E^bind^* increased with *θ* from 0° to 90°, which quantitatively agreed with the results of the experiments. The maximum binding energy was at the edge dislocation, and the value, *E^bind^* = 0.42 eV, was quite consistent with other atomistic studies [24]. The minimum binding energy was at the screw dislocation, and the value, *E^bind^* = 0.26 eV, was in the range of the experimental estimations, i.e., 0.21 eV~0.31 eV [26,27]. As a comparison, the H binding energy obtained by different experimental methods is shown in Table 2. It can be seen that depending on the applied method, the H binding energy could vary widely from 0.21 eV/H to 0.62 eV/H, and most past studies have agreed with an H binding energy of ~0.28 eV/H even though it is difficult to determine the type of dislocation. This value was very close to the binding energy of H with a screw dislocation (0.26 eV), which may have been due to the fact that more screw dislocations could be generated because of the lower formation energy as compared to mixed and edge dislocations. Moreover, it was interesting to find that *E^bind^* increased linearly with *θ* in the region of *θ* ≥ 10°, and the calculated increment was approximately 1.1 meV/deg.

### 3.2. The Effect of H on the Dislocation Energies

The atomistic results of the dislocation line energies *E* in the presence of H were calculated by:

(2) $$E=\frac{1}{l}(∆E_{i,Fe}+∆E_{j,H})$$

where Δ*E_i_*_,*Fe*_ is the energy difference of the *i*th H between the states around the dislocation and at the tetrahedral site and Δ*E_i_*_,*Fe*_ is the energy difference of the *j*th Fe between the states in the dislocation box and those in the perfect Fe lattice. The final dislocation line energy was the average value of over 100 equilibrium microstates generated by the GCMC simulations.

**Figure 3:**
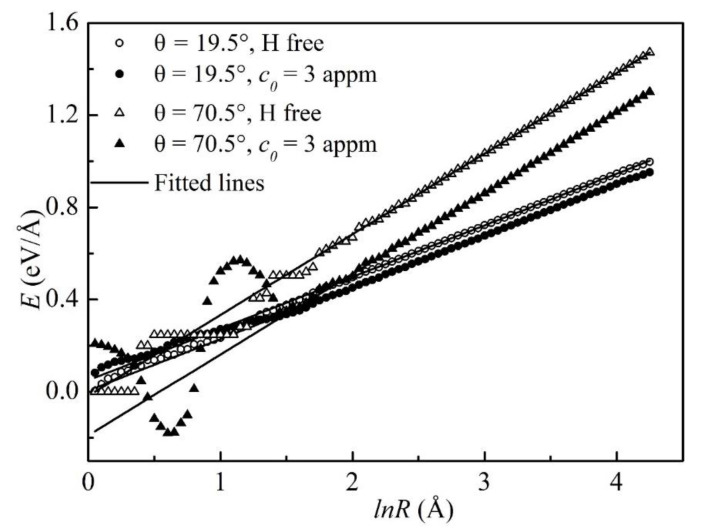
Dislocation line energies as functions of the logarithmic *R* at *c_0_* = 0 and 3 appm for the 19.5° and 70.5° dislocations.

As an example, the atomistic line energies of the 19.5° and 70.5° dislocations as functions of *lnR* are shown in Figure 3. As expected from the elastic theory, the atomistic energies varied linearly with *lnR* in the region outside the core. The fitted lines to the *E*~*lnR* data in the linear region based on the elastic theory are also shown in Figure 3 as solid lines. Δ*E* was defined as:

(3) $$∆E=E-E^{H}$$

where *E^H^* and *E* are the dislocation line energies of the H-charged and the H-free dislocations, which were computed from the fitted linear functions. With such a definition, a positive Δ*E* indicated a reduction in the dislocation line energy while a negative value indicated an increase in the dislocation line energy.

The H-induced changes in the dislocation line energies are listed in Table 3. These were consistent with the predictions of the defactant theory, according to which H is considered as a defect and decreases the formation energies of dislocations [28]. The decrement in the line energy increased with *θ* (or the edge component) since more H atoms were trapped around the edge dislocations because of the stronger H binding energy, as suggested in Figure 2. This was agreement with the study of Sills et al. [29] on Ni quantitatively, where the energy reduction in a dislocation induced by H was computed using a continuum mode. The results showed that the reduction in the dislocation core energy increased with the character angle *θ* due to the formation of hydrides at the cores. Although only the core energy that changed as a function of *θ* was computed in their work, the identical dependence of the decrement in the dislocation line energy on the character angle could be expected. The H concentration was much lower than that in the Ni, and no hydrides were formed at the cores. We continued to observe that the decrement in the line energy increased with *θ*, in keeping with their results.

### 3.3. H Atmosphere around the Dislocations

**Figure 4:**
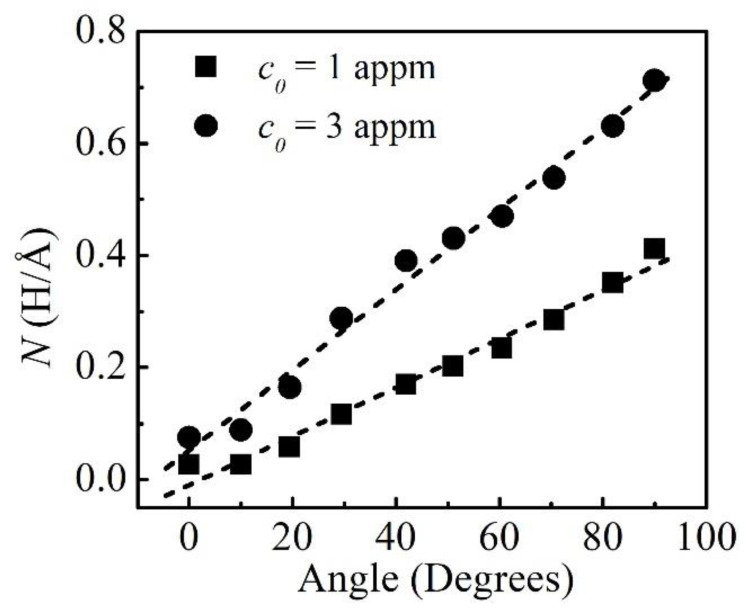
Excess H as a function of *θ* at *c_0_* = 1 and 3 appm. The symbols are the atomistic simulations and the dotted lines are the trend lines.

The H atmosphere around the dislocations was studied based on the calculations of the excess H, and the atomistic results were computed as the average value from the trapped H number over 50,000 GCMC cycles after the H concentration reached equilibrium. Since the H concentrations in the elastic fields of the dislocations were extremely low, it was reasonable to consider the excess H *N* within *R* < 8 nm as the total excess H around the dislocations. The obtained *N* values at various bulk H concentrations *c_0_* were as shown in Figure 4. It was evident that *N* increased sharply with *θ*. It could also be seen that *N* nearly increased linearly with *θ* at small *c_0_* values.

## 4. Discussions

### 4.1. The Interaction Energy of H

It is well-known that the decrement in the line energy depends on the interaction energy and the excess H [30,31]. The interaction energy *E^int^* is thus defined as a reduction in the dislocation line energy induced per H-atom, i.e.,

(4) $$E^{int}=\frac{∆E}{N}$$

**Figure 5:**
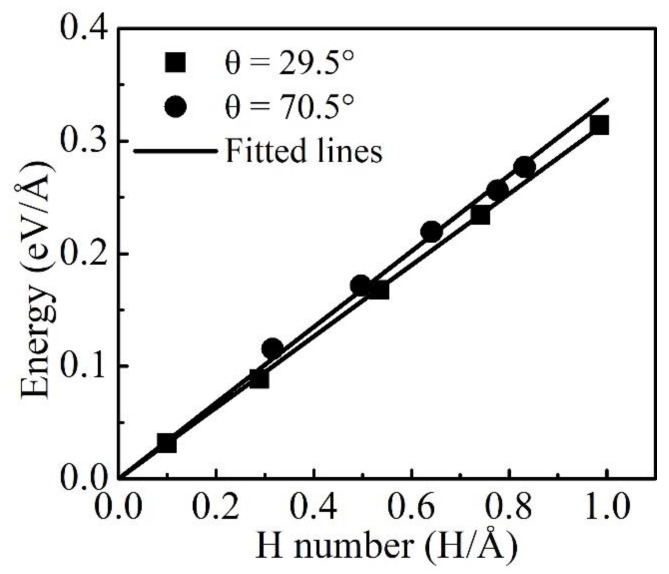
H-induced reductions in the dislocation line energies as functions of the excess H for the 29.5° and 70.5° dislocations.

Here, *N* is the excess H and is averaged from the obtained H numbers over the 100 microstates that were used to evaluate the dislocation line energy in Section 3.2. If ignoring the multiple H–H interactions, it can be expected that *E^int^* should be equal to *E^bind^*, and therefore, Δ*E* would increase linearly with *N*. As shown in Figure 5, Δ*E* typically varies linearly with *N*. The *E^int^* was evaluated as the slopes of the fitted lines to the Δ*E*~*N* data, and the obtained results are shown in Figure 2. It can clearly be seen that there was a decrease in *E^int^* as compared to *E^bind^* for *θ* ≥ 10°, and the decrement in *E^int^* increased with *θ*, which could be attributed to the repulsive interactions among the multiple H-atoms near the mixed and edge dislocation cores. It can also be seen that there were no differences between *E^int^* and *E^bind^* for *θ* = 0° because of the small excess H around the screw dislocation. A linear dependence of *E^int^* on *θ* in the region of *θ* ≥ 10° was observed. The increment in *E^int^* from 10° to the edge dislocation was 0.7 meV/deg, which was smaller than that for *E^bind^* of 1.1 meV/deg.

### 4.2. The Effect of H on Slip Planarity

The influence of hydrogen on slip planarity was studied based on the energy changes in the system when an edge dislocation was converted towards a screw dislocation [32]. In the presence of H, the energy changes in the system *dE^H^* were defined as the energy differences between the initial and target dislocations, as follows:

(5) $$dE^{H}=(E_{1}-N_{1}E_{1}^{int})-(E_{2}-N_{2}E_{2}^{int})$$

where *E_i_*_=1,2_ represents the line energies of the initial and target dislocations in the absence of H, $E_{i=1,2}^{int}$ represents the interaction energies of H with the dislocations, and *N_i_*_=1,2_ the excess H around the initial and target dislocations. The term $N_{i}E_{i}^{int}$ denotes the reductions in the dislocation line energies induced by H. In the absence of H, the energy change can simply be calculated as $dE=E_{1}-E_{2}$. The difference in the energy change of the system is given by:

(6) $$\delta E=N_{1}E_{1}^{int}-N_{2}E_{2}^{int}$$

With such a definition, a positive *δE* indicated that there was an increment in the energy change in the system with H. Clearly, *δE* was determined by the reductions in the dislocation line energies.

**Figure 6:**
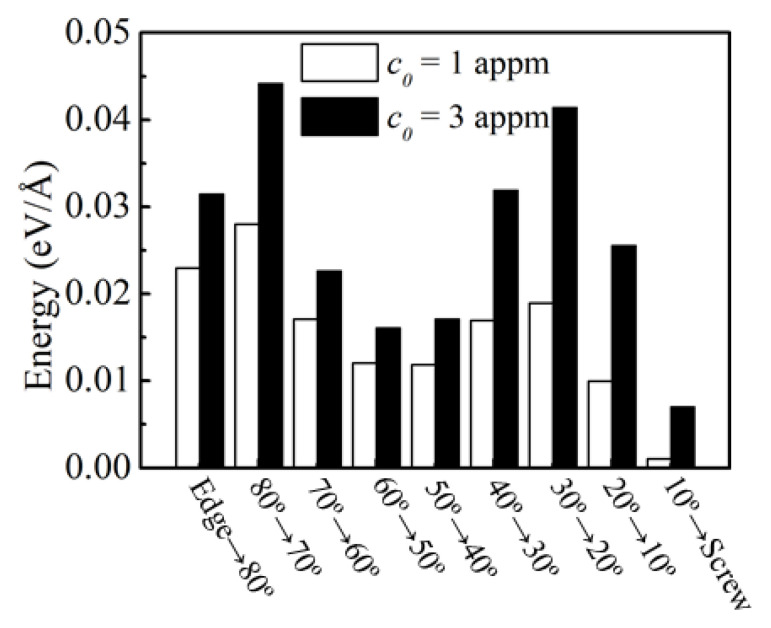
*δE* values for various conversion types at *c_0_* = 1 and 3 appm.

We considered the condition that when slipping was impeded, the edge and mixed dislocations were forced to reorient towards screw dislocations gradually, i.e., edge → 80°, 80° → 70°, 70° → 60°, …, and 10° → screw, and then the screw dislocation would proceed to move by cross-slip. This reorientation process led to a decrease in the interaction energy of H (*E^int^*) and the excess H in the atmosphere (*N)*. The energy differences (*δE)* during this reorientation process were calculated according to Equation (6), and the obtained results are shown in Figure 6. It was clear that all the values were positive, suggesting that H increased the energy changes in the system as expected from the mechanism of the edge dislocation energy reduction in the slip planarity, even though the imposed H concentrations were only 1~3 appm in our simulations. Thus, *δE* would suppress the formation of screw dislocations and then decrease the tendency to cross-slip, assisting the slip planarity in the bcc Fe.

## 5. Conclusions

We completed a systematic study on the interaction between H and ½ <111> {110} dislocations using the EAM potential for an Fe-H system. The binding energy of H, the excess H in the atmosphere, and the interaction energy of H for 10 types of dislocations were evaluated and analyzed, and then the effect of H on the slip planarity in the bcc Fe was discussed. The conclusions were as follows:

(1). The binding energies of H, the excess H in the atmosphere, and the decrements in the dislocation line energies were increasing with the edge components.
(2). The interaction energy of H was slightly weaker than its binding energy because of the multiple H–H interactions.
(3). H induced a significant increment in the energy change in the system, resulting in the inhibition of the reorientations towards the screw dislocations from the edge and mixed dislocations. The cross-slip in the bcc Fe was thus decreased, facilitating slip planarity.

## Figures and Tables

**Table 1:** Orientations of the simulation box containing dislocations with various character angle values of *θ*.

*θ* (Degrees)	*x*	*y*	*l//z*
90.0	[111]	$\left[\bar{1}10\right]$	$\left[\bar{1}\bar{1}2\right]$
82.0	$\left[\bar{3}\bar{3}\bar{4}\right]$	$\left[\bar{1}10\right]$	$\left[22\bar{3}\right]$
70.5	$\left[\bar{1}\bar{1}\bar{2}\right]$	$\left[\bar{1}10\right]$	$\left[11\bar{1}\right]$
60.5	$\left[\bar{1}\bar{1}\bar{3}\right]$	$\left[\bar{1}10\right]$	$\left[33\bar{2}\right]$
51.1	$\left[\bar{1}\bar{1}\bar{5}\right]$	$\left[\bar{1}10\right]$	$\left[55\bar{2}\right]$
42.0	$\left[\bar{1}\bar{1}\bar{16}\right]$	$\left[\bar{1}10\right]$	$\left[88\bar{1}\right]$
29.5	$\left[11\bar{14}\right]$	$\left[\bar{1}10\right]$	[771]
19.5	$\left[11\bar{5}\right]$	$\left[\bar{1}10\right]$	[552]
10.0	$\left[11\bar{3}\right]$	$\left[\bar{1}10\right]$	[332]
0.0	$\left[11\bar{2}\right]$	$\left[\bar{1}10\right]$	[111]

**Table 2:** H binding energy with the dislocations obtained from the experiments.

*E^bind^* (eV/H)	Method
0.27, 0.28	Temperature dependence in H diffusivity
0.28	Thermal spectrum of H_2_ evolution
0.50–0.60, 0.62	Analysis of permeability
0.21, 0.28	Internal friction

**Table 3:** The hydrogen-induced changes in the dislocation line energy Δ*E* as a function of *θ*.

*θ* (Degrees)	Δ*E* (eV/Å)		
	1 appm	3 appm	5 appm
90.0	0.126	0.292	0.297
82.0	0.149	0.210	0.244
70.5	0.115	0.171	0.219
60.5	0.078	0.158	0.170
51.1	0.076	0.145	0.147
42.0	0.055	0.149	0.149
29.5	0.032	0.088	0.168
19.5	0.029	0.045	0.085
10.0	0.002	0.022	0.028
0.0	0.005	0.005	0.027

## Data Availability

No new data were created or analyzed in this study. Data sharing is not applicable to this article.
